# Delta Describe, the French Collaborative Project: The Profile and Management of Hepatitis Delta Patients in Metropolitan France

**DOI:** 10.3390/v18040424

**Published:** 2026-03-31

**Authors:** Marie Bosselut, Paul Carrier, Ségolène Brichler, Sophie Alain, Marilyne Debette-Gratien, Caroline Scholtès, Anne-Marie Roque-Afonso, Sonia Burrel, Pascale Trimoulet, Aurélie Guigon, Marianne Coste-Burel, Eric Billaud, Jacques Izopet, Karine Saune, Stéphane Chevaliez, Benoit Visseaux, Anaïs Soares, Jean-Pierre Bronowicki, Jérôme Boursier, André-Jean Remy, Vincent Quentin, Isaac Fassler, Bernard Castan, Gérard Lina, Cécile Brouard, Katell Peoc’h, Hélène Fontaine, Marc Bourlière, Dominique Roulot, Dadi Abel Diédhiou, Céline Rigaud, Sandrine François, Véronique Loustaud-Ratti, Delta Describe Study Group

**Affiliations:** 1 Hepato-Gastroenterology Department, Limoges University Hospital Center, 87042 Limoges, France; 2 Virology Laboratory, Avicenne Hospital, AP-HP, 93000 Bobigny, France; 3 Virology Department, Limoges University Hospital Center, 87042 Limoges, France; 4 Virology Laboratory, Institut des Agents Infectieux, HCL, 69004 Lyon, France; 5 Virology Laboratory, Paul Brousse Hospital, AP-HP, 94804 Villejuif, France; 6 Virology Laboratory, Bordeaux University Hospital Center, 33000 Bordeaux, France; 7 Virology Laboratory, Lille University Hospital Center, 59000 Lille, France; 8 Virology Laboratory, Nantes University Hospital Center, 44093 Nantes, France; 9 Department of Infectious Diseases, Nantes University Hospital Center, 44093 Nantes, France; 10 Virology Laboratory, Toulouse University Hospital Center, 31059 Toulouse, France; 11 Virology Laboratory, Henri Mondor Hospital, AP-HP, 94010 Créteil, France; 12 Laboratoire Cerba, 95740 Frépillon, France; 13 Department of Infectiology, Eurofins Biomnis, 69007 Lyon, France; 14 AFEF, French Association for the Study of the Liver, 75006 Paris, France; 15 ANGH, National Association of Hepato-Gastroenterology Groups, 93370 Montfermeil, France; 16 SFHGL, French Society of Hepatology and Gastroenterology, 54600 Villers-Lès-Nancy, France; 17 SPILF, French Society of Infectious Diseases, 75010 Paris, France; 18 SFM, National Society of Microbiology, 75014 Paris, France; 19 SPF, Public Health France, 94410 Saint-Maurice, France; 20 SFBC, French Society of Clinical Biology, 75006 Paris, France; 21 University of Paris City, Beaujon, AP-HP, INSERM UMR 1149, 92110 Clichy, France; 22 Department of Hepatology and Gastroenterology, Hopital Saint-Joseph, 13008 Marseille, France; 23 Hepatology Unit, Avicenne Hospital, University of Paris, 93000 Bobigny, France; 24 Research and Clinical Data Center (CDCR), Limoges University Hospital Center, 87000 Limoges, France

**Keywords:** hepatitis delta virus, real-world study, bulevirtide, liver fibrosis, France

## Abstract

Hepatitis delta (HDV) infection affects 5% of hepatitis B (HBV)-positive patients and is associated with an increased risk of cirrhosis and hepatocellular carcinoma; however, it remains underdiagnosed. The first part of our Delta Describe study highlights the insufficient level of HDV screening among patients in metropolitan France. In this study, we report on their real-world management. Patients with at least one positive HDV RNA test performed in 2019 were identified through the major public and private laboratories in France. From January 2024 to July 2025, informed patients were interviewed, and physicians supplemented the collected data. A total of 547 patients were included, with a median age of 44 years; most originated from Africa or Eastern Europe. HIV and hepatitis C coinfections were reported in 15.2% and 4.6% of patients, respectively. Liver fibrosis was primarily assessed using FibroScan^®^. Most patients knew the year of their delta diagnosis, and 69.1% knew their fibrosis stage. Liver-related events occurred in 14.3% (67/468) of patients, mainly comprising portal hypertension (61.6%), liver failure (12.3%), and hepatocellular carcinoma (26%), and 45 patients (45/468) underwent liver transplantation. At the time of the survey, 47.1% of the patients reported undetectable HDV RNA; 40.6% (222/547) had currently or previously undergone BLV treatment. Among patients receiving ongoing treatment for HDV at the time of the survey, 84.8% were receiving nucleos(t)ide analogs (NUCs). In metropolitan France, HDV patients had access to specialized follow-up care and innovative therapies (bulevirtide), were mostly on NUCs, and demonstrated good disease awareness.

## 1. Introduction

According to the World Health Organization (WHO), hepatitis B virus (HBV) affects more than 250 million people worldwide, approximately 5% of whom also carry hepatitis delta virus (HDV) [1]. In Stockdale’s meta-analysis, performed across six WHO regions, the estimated prevalence of anti-HDV antibodies (HDV-Ab) was 4.5% (2.10–6.28) in the population positive for HBV surface antigen (HBs-Ag). The highest prevalence was detected in Mongolia, the Republic of Moldova, and countries in Western and Middle Africa, and in the following populations: injecting drug users, men who have sex with men, sex workers, and people infected with hepatitis C virus (HCV) or human immunodeficiency virus (HIV) [2]. In 2015, a French study synthesized data from several cohorts and reported a low prevalence of HDV infection in France, with a carriage rate of HDV-Ab of 4% in HBV-positive patients, mainly originating from high- or medium-endemic countries [3]. Finally, according to the Polaris-adjusted estimate of the prevalence of HDV in France, 3800 patients are HDV RNA+, with 28.7 screening tests required to diagnose one case [4].

HDV, the smallest known single-stranded circular RNA virus, does not encode its own envelope proteins and depends on the expression of HBs-Ag to enter hepatocytes and subsequently assemble new HDV viruses [5]. HDV infection, which results from superinfection in 80% of cases, is a serious disease and a major public health problem, even in France. A French retrospective study conducted by the National Reference Center (NRC), based on targeted data from 1112 HDV patients referred to university hospital centers, showed that 312 patients (28.2%) had cirrhosis at referral. The median age of HDV patients was 36.5 years, with a male predominance (68.6%) [6]. A comparison of two large French cohorts of HDV and HBV cirrhotic patients revealed that the cumulative incidence of hepatocellular carcinoma (HCC) at 1, 3, and 5 years was 5.2%, 11.8%, and 20.2% for HDV patients versus 1.1%, 2.5%, and 4.4% for HBV patients, respectively. The cumulative incidence of liver decompensation was 5.0%, 13.3%, and 18.8% for HDV patients versus 1.2%, 3.3%, and 4.7%, for HBV patients, respectively [7].

Currently, different international guidelines [8] and the French National Authority for Health (HAS) [9] recommend systematic screening for HDV-Ab in the event of any new positive HBs-Ag findings, which should be repeated in the presence of persistent risk factors or unexplained elevations in ALT levels [10].

The results of the first part of our Delta Describe study, which assessed HDV screening in metropolitan France from 2016 to 2022 using the French National Health Data System (SNDS), clearly showed that screening had improved (multiplied by 2.3), but remained insufficient [11].

A majority of university hospital laboratories in France perform reflex testing, which may have reduced the time to diagnosis and improved access to care for patients [12]. However, private laboratories do not perform reflex testing, partly due to a lack of reimbursement for tests in this context, and partly because of the subcontracting of anti-HDV-Ab testing. Facilities serving migrant populations use point-of-care dried blood spot tests for the systematic screening of HBV, hepatitis C (HCV), HIV, and more recently HDV, which is currently under validation.

Until 2019, pegylated interferon (PEG-IFN) alpha was the only treatment available for patients with HDV. Since relapses were frequent after stopping treatment [13], mild-to-moderate tolerance problems, which can easily be managed during limited treatment with PEG-IFN, were more difficult to manage over long periods [14]. Bulevirtide (BLV), an entry inhibitor of HBV/HDV, which was first made available in France in 2019 through an early access program and was then granted marketing authorization in 2020, has opened up new therapeutic perspectives to limit the incidence of HDV complications [15]. It is administered as monotherapy or in combination with PEG-IFN [16], with the latter being recommended by the HAS in the absence of contraindications [17]. In a real-world study, a combined response (virological and biochemical) to long-term bulevirtide therapy, at weeks 48 and 96, was observed in 44% and 54% of patients with compensated cirrhosis, respectively [15]. Finite therapy of 2 mg BLV, in combination with PEG-IFN, led to a significant durable response, with 26% of patients achieving HDV RNA undetectability off therapy [16], but the continued search for new finite therapies remains necessary.

Few data are available in France regarding access to BLV, a treatment with daily injections, among a population that is often young and unstable, and faces significant language barriers and challenges in maintaining long-term medical follow-up. In this second part of the Delta Describe study, we focus on the real-world management of delta patients in metropolitan France.

## 2. Methods

The main objective of this study was to describe the management of patients who underwent an HDV RNA + test in 2019 and were followed up until 2025, and to specify their epidemiological characteristics and the prescriber profile.

### 2.1. Data Sources

Seven public university hospital laboratories (Lille, Lyon, Nantes, Toulouse, Paul Brousse, Henri Mondor, and Avicenne) and two groups of private laboratories (Cerba and Eurofins Biomnis), using HDV PCR and covering most of the metropolitan territory, agreed to participate in the study. We were able to retrieve the total number of HDV RNA tests carried out in 2019; the identity, sex, date of birth, and telephone number of each patient; and the name of the prescriber, thanks to the lists of patients provided by the laboratories. Using these data, we then contacted, by phone, either the patients themselves, their practitioner, or both, to answer a questionnaire for data collection (Table 1).

### 2.2. Ethical Considerations

This is a national, multicenter, human-participant study using personal data registered on ClinicalTrials.gov (identifier: NCT05936073). This study received approval from the North-West II Committee for the Protection of Persons and complies with the CNIL’s (French National Commission on Informatics and Liberty) “reference methodology”. Laboratories with patient contact information sent an information letter to each patient, in accordance with the General Data Protection Regulation. Individuals had one month to refuse to participate in the research by sending back an objection form.

After this period, the lists of patients who did not object were transmitted via our secured platform Pydio 7 (pydio.com) at Limoges University Hospital. The patients’ identities were used to contact them and to complete the questionnaires, but the data were then pseudonymized when entered in the eCRF (Electronic Case Report Form) (Ennov Clinical CSOnline, V8.0.120). The data were collected from January 2024 to July 2025.

### 2.3. Statistical Analysis

Data were extracted and analyzed by the CDCR (Clinical and Research Data Center) of the Limoges University Hospital using SAS Enterprise Guide v7.1.0. Categorical results were reported as numbers and percentages, while quantitative variables were described using the median and interquartile range (IQR). Statistical comparisons were performed using a chi-square test. Univariate analyses were first performed to identify variables associated with the outcome of interest, and variables with potential associations (*p* < 0.2) were subsequently included in a multivariate model. A backward variable selection procedure was then applied in the multivariate analysis, with a significance threshold of *p* < 0.05. A *p*-value < 0.05 was considered statistically significant.

## 3. Results

### 3.1. Selection of Patients

**Figure 1:**
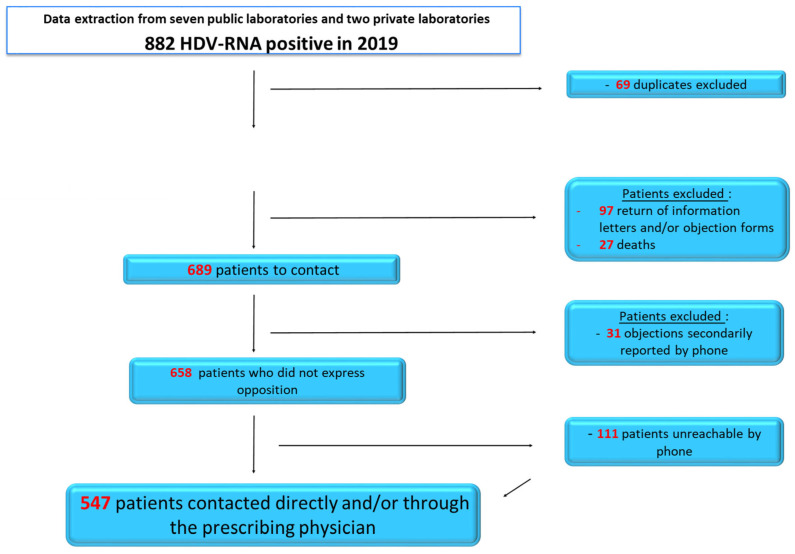
Patient flow diagram.

Among the 882 HDV RNA-positive tests identified in 2019, duplicates (repeated tests in different laboratories) were removed. Then, deceased patients (identified from https://www.deces-en-france.fr/ accessed on 1 April 2024), patients who returned the information letters and/or objection forms, and patients who later declined to participate in the study were excluded. Of the 689 patients contacted, 111 remained unreachable. In total, the data from 547 patients were successfully collected (Figure 1).

### 3.2. Characteristics of the Population

**Figure 2:**
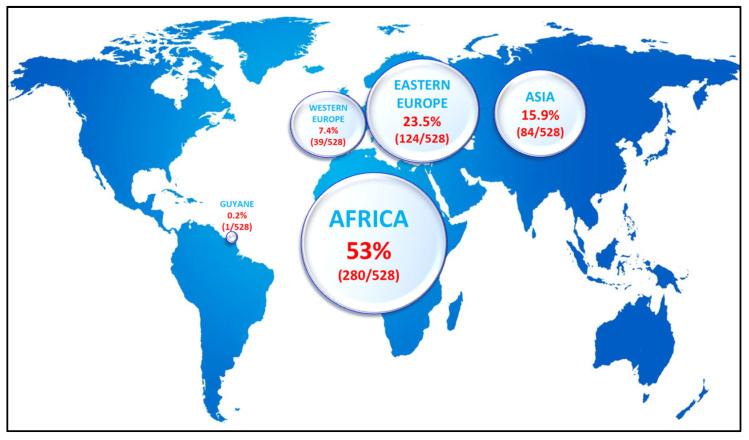
Geographical origin of the patients included in the study (data available for 528/547 patients).

The median age was 44 years old [IQR, Interquartile Range 37–53] and the sex ratio (male/female) was 0.56. The year of diagnosis was known for 88.1% of patients, and the median duration of disease from diagnosis to the time of interview was 9 years [IQR 6–16]. Concerning co-infections, 15.2% of patients reported HIV and 4.6% HCV co-infections, while 3.8% were unaware of their co-infection status. Among the 528 patients who reported their native country, 53% (280/528) were of African origin, 23.5% (124/528) were from Eastern Europe, 7.4% (39/528) were from Western Europe, and 15.9% (84/528) of Asian origin, of whom 67.9% were born in Mongolia (Figure 2).

**Figure 3:**
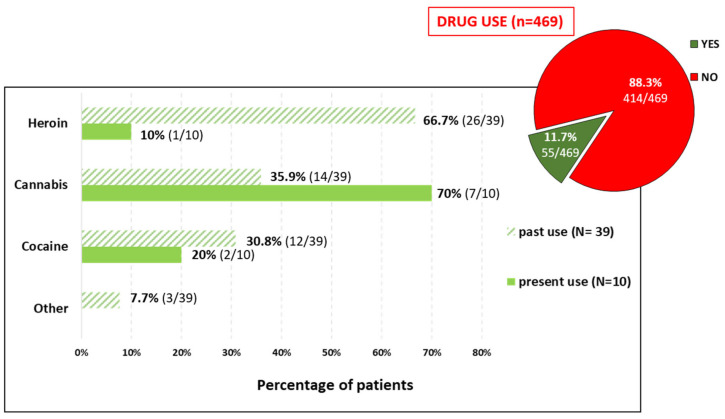
Type of drug use (current and past) by patients for whom data were available. Two patients with past use and two patients with current use did not report the type of drug used.

Among the 469 patients who answered the question(s) about drug use, 11.7% (55/469) reported currently using, or having used, drugs. This mainly related to past drug use. The drugs reported by active users (*N* = 10) were mainly cannabis (70%), cocaine (20%), and heroin (10%) (Figure 3).

### 3.3. Patient Care Pathway and Follow-Up

**Figure 4:**
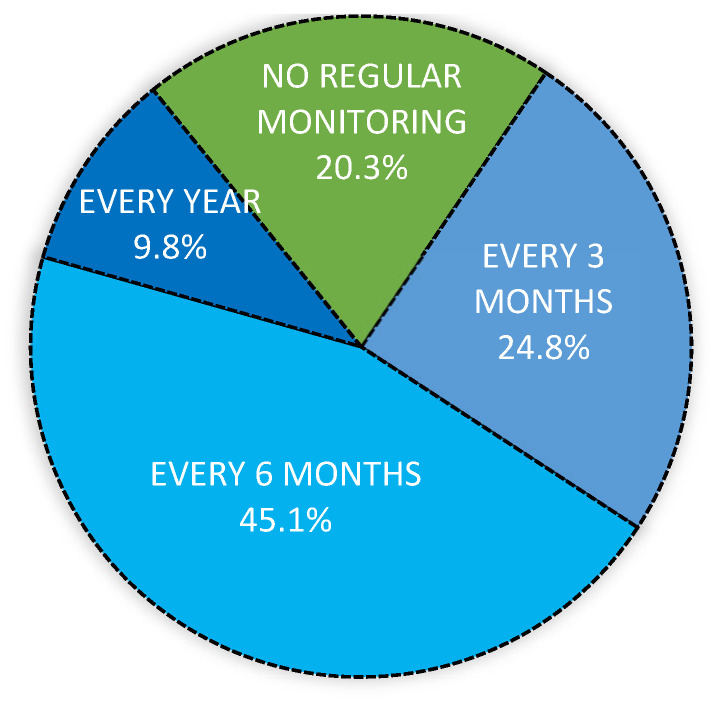
Frequency of follow-up visits (data available for 533/539 patients).

At the time of the initial diagnosis of HDV, 98.7% (539/546) of patients were referred to a specialist physician. At the time of the interview, most patients (79.7%, 425/539) still benefited from specialized follow-up care at least once a year, 24.8% (132/533) every 3 months, 45.1% (241/533) every 6 months, and 9.8% (52/533) once a year (Figure 4). The practitioners involved were hepato-gastroenterologists in 81% of cases, infectious disease specialists in 12% of cases, and a specialist in another area (hematologist, oncologist, etc.) in 7% of cases. Of the 475 patients for whom this information was available, 78.3% (372/475) reported having a general practitioner for overall management.

### 3.4. Level of Fibrosis

**Figure 5:**
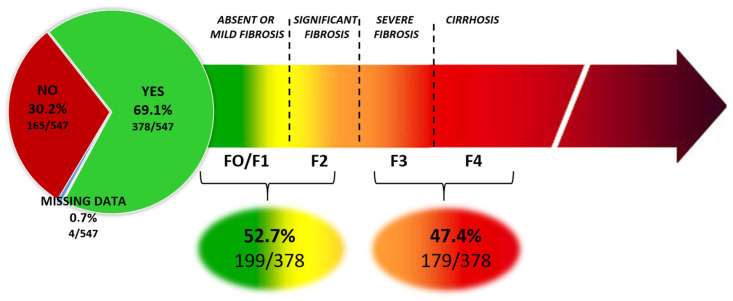
Reported fibrosis stage by patients and/or physicians at the time of the survey (N = 378/547): F0/F1/F2 (N = 199); F3/F4 (N = 179).

Fibrosis was assessed in 75.3% (412/547) of patients, mainly using Fibroscan^®^ (99%, 406/410), and 52.8% (289/547) had previously undergone a liver biopsy (between 1982 and 2024). We were able to collect the most recent fibrosis score for 378 patients (69.1%, 378/547). When the LSM value was available, we used the thresholds recently published by Roulot et al. [18]: advanced fibrosis is diagnosed when the LSM value is greater than 10 kPa, and cirrhosis when the LSM value is greater than 12 kPa. The stages of fibrosis, evaluated using Fibroscan^®^ and/or reported by patients or physicians at the time of the interview, were classified as absent-to-moderate fibrosis (F0/F1/F2) for 52.7% (199/378) and advanced fibrosis for 47.4% of patients (179/378) (Figure 5). Among the 179 patients with declared advanced fibrosis, cirrhosis was suspected in 153 patients (85.5%).

Taking into account the potential relationship between patients’ fibrosis stage and the following variables, sex, age, and migrant origin, we found in the final multivariable model that the risk of advanced fibrosis was lower in patients of African ancestry, independent of age (age < 40 years: aOR [0.18 0.08–0.39], *p* = 0.0001; age ≥ 40 years: OR [0.54 0.32–0.93], *p* = 0.0264; see Supplementary Table S1).

### 3.5. Liver Complications

In total, 102 patients reported 136 complications related to their condition. Given that transplant recipients had difficulty describing the nature of the complications that led to the transplant, we focused on the 67 non-transplant recipients who reported 73 complications.

**Figure 6:**
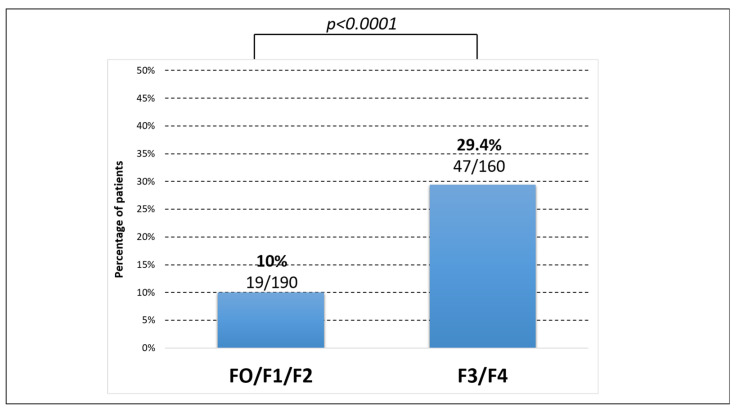
Percentage of liver complications according to the presence or absence of advanced fibrosis at the time of the survey. F0/F1/F2 = 190; F3/F4 = 160. The distribution of complications according to fibrosis score was established based on the number of patients who had both a fibrosis assessment and data complications (N = 350).

Patients classified as F0/F1/F2 fibrosis had significantly fewer complications than those classified as F3/F4 (*p* < 0.05) (Figure 6).

Considering the potential relationship between patients’ liver complications and demographic and clinical variables, in the final multivariable model, we observed that patients with advanced fibrosis F3/F4 were more likely to develop liver complications (adjusted OR = 3.74; 95% CI [2.09–6.71]). Gender, age, and migrant origin were not associated with the occurrence of liver complications (see Supplementary Materials Table S2).

### 3.6. Decompensated Cirrhosis and CHC

Among the 468 patients whose data were available, 45 patients underwent liver transplantation, but the underlying complications leading to transplantation could rarely be reported.

Excluding patients who underwent liver transplantation, 14.3% (67/468) of the patients experienced decompensated cirrhosis or CHC: 62.7% of the patients (42/67) had one, 25.4% (17/67) two, 10.5% (7/67) three and 1.5% (1/67) four complications.

**Figure 7:**
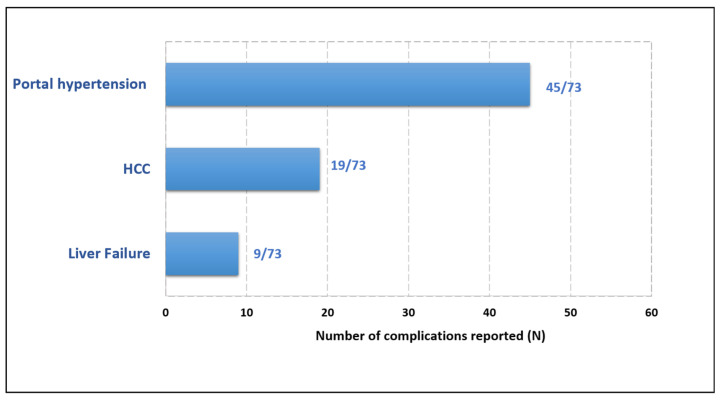
Complications reported by the 67 patients, excluding liver transplant recipients (number of complications: 73). Portal hypertension includes ascites, hepatorenal syndrome, and esophageal or gastric varices with or without gastrointestinal bleeding. HCC = hepatocellular carcinoma. Liver failure includes jaundice, hepatic encephalopathy, and severe or fulminant hepatitis.

Among the 73 serious complications reported (Table 2), 61.6% (45/73) were related to portal hypertension (ascites; esophageal or gastric varices, with or without gastrointestinal bleeding; and hepatorenal syndrome), 12.3% (9/73) were attributed to liver failure (jaundice, hepatic encephalopathy, or severe/fulminant hepatitis), and 26% (19/73) to hepatocellular carcinoma (Figure 7).

### 3.7. HDV and HBV Viral Loads

In this study, 73.1% (400/547) of the surveyed patients knew whether their current HDV RNA status was positive or negative, compared to 71.5% (391/547) for HBV DNA. Among them, 52.9% (211/400) had a positive HDV RNA result and 31.4% (122/389) had detectable HBV DNA, respectively. The participating laboratories were surveyed regarding the molecular biology techniques employed for RNA testing in 2019. Of the nine laboratories, seven (77%) used the EurobioPlex HDV qRT-PCR EBX-004 (Eurobio Scientific, Les Ulis, France), with an LOD/lower limit of quantification (LOQ) of 100 IU/mL [19], and two (23%) used an “in-house” technique.

### 3.8. Ongoing Treatments

**Figure 8:**
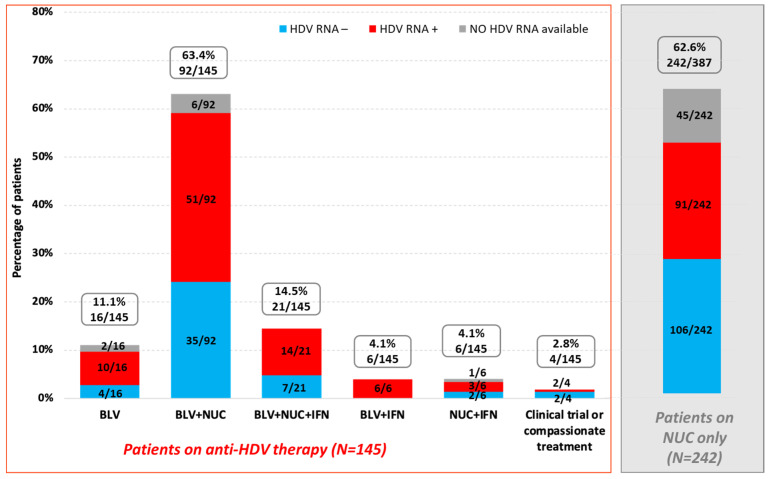
Current anti-HBV and HDV treatments of patients at the time of the survey according to HDV RNA status (total number of patients who provided the information: N = 387).

Among the 477 patients for whom this information was available, 81.1% (387/477) were receiving treatment for HDV and/or HBV. Specifically, 37.5% (145/387) of patients were undergoing anti-HDV therapy. Among them, 93.1% (135/145) were receiving BLV with or without PEG-IFN, 4.1% were receiving PEG-IFN +NUC (6/145), and 2.8% were receiving (4/145) treatment as part of a clinical trial or via compassionate use (SOLSTICE trial, lonafarnib/ritonavir). Among patients who tested positive for HDV RNA in 2019, 93.3% (361/387) were receiving NUC therapy at the time of the survey; 62.6% (242/387) of patients undergoing antiviral therapy were taking NUCs only (Figure 8).

Among patients currently on bulevirtide (N = 135), 63.7% (86/135) reported having previously received HDV and/or HBV treatment; more specifically, 95.3% (82/86) had received anti-HDV therapy, primarily PEG-INF with or without NUC (84.9%, 73/86), and 9.3% (8/86) had received bulevirtide, with or without PEG-INF or NUC. A total of 49 patients were unable to provide information on the previous treatment they had received.

We examined the HDV viral load results (HDV RNA+ vs. HDV RNA−) of the 145 patients on anti-HDV therapy, depending on the type of ongoing treatment. At the time of data collection, excepting patients for whom no HDV RNA data were available, 63.2% (86/136) were still replicating (with 94.2% (81/86) of them being on BLV with or without PEG-IFN), and 36.8% (50/136) had undetectable HDV RNA (with 92% (46/50) of them being on BLV with or without PEG-IFN). Among the 197 patients who received NUCs only and had HDV RNA available, 46.2% (91/197) were HDV RNA-positive (Figure 8).

### 3.9. Past Treatments

**Figure 9:**
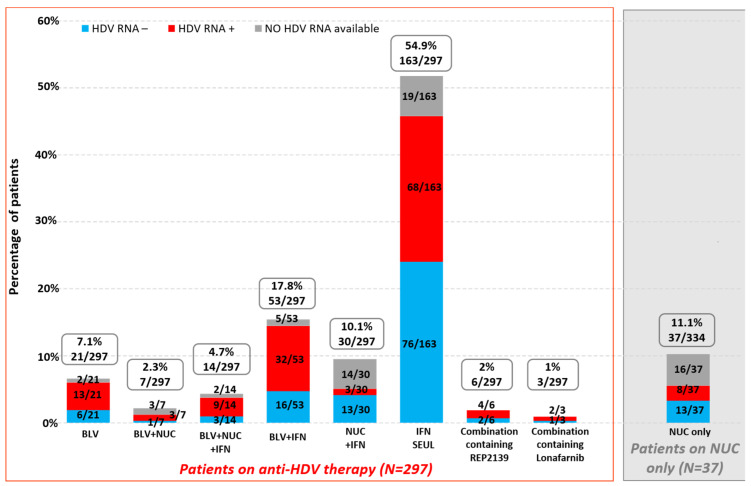
Past anti-HBV or HDV treatments of patients at the time of the survey according to HDV RNA status (data available for 334/336 patients).

Among the 443 patients for whom this information was known, 75.8% (336/443) had previously received treatment for hepatitis D and/or hepatitis B, and treatment information was obtained for 334 patients. Specifically, 88.9% (297/334) of patients had undergone anti-HDV therapy: PEG-IFN alone was the most frequent regimen (54.9%, 163/297), followed by BLV with or without PEG-IFN (32%, 95/297). In addition, 3% (9/297) of patients had received REP2139 or lonafarnib with or without PEG-IFN, after BLV failure. Finally, 11.1% (37/334) of patients had received NUCs only (Figure 9).

We examined the current HDV viral load results (HDV RNA+ vs. HDV RNA-) of the 297 patients who had previously undergone anti-HDV treatment. We observed that 53.2% (134/252) were still replicating at the time of data collection, with the exception of patients for whom no data on HDV RNA were available (57/134, 42.5% of them had received BLV with or without PEG-IFN), and 46.8% (118/252) had an undetectable HDV RNA (76/118 of them had received only PEG-IFN and were on BLV after interferon failure) (Figure 9).

In summary, 40.6% (222/547) of the patients included in the study had a declared current or prior BLV. Two hundred and two patients had an informed HDV RNA status of whom 35.2% (71/202) were RNA-negative and 64.8% (131/202) RNA-positive at the time of the survey.

**Figure 10:**
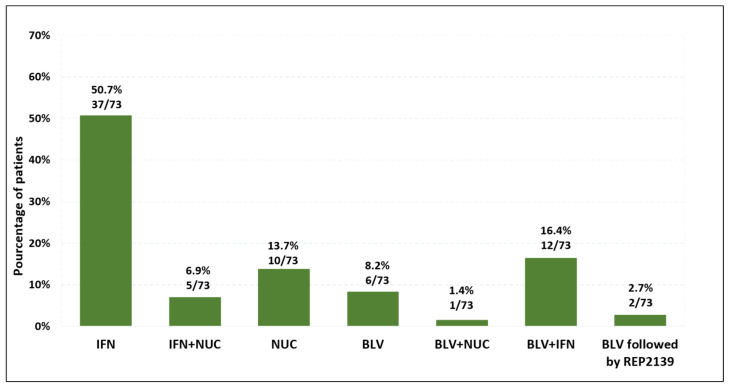
Previous hepatitis delta treatments in patients HDV RNA-negative and receiving NUCs at the time of the questionnaire (excluding transplant recipients, N = 73).

Finally, among the 93 HDV RNA-negative patients treated only with NUCs at the time of the questionnaire (excluding transplant recipients), 74 patients had declared having had hepatitis delta treatments (79.6%) with one missing information regarding the type of treatment. (Figure 10). The majority had previously received PEG-IFN alone (50.7%, 37/73), or BLV alone (8.2%, 6/73) or BLV combined with PEG-IFN (16.4%, 12/73), or BLV followed by REP2139 after BLV failure (2.7%, 2/73). Notably, 10.8% (10/93) never received any specific treatments for HDV and nine reported not knowing whether they had received any prior treatment (9.7%).

We examined the potential associations between previous or current bulevirtide exposure and clinical, as well as demographic, characteristics, and identified a significant interaction between geographic origin and fibrosis stage (*p* = 0.0022). In the final multivariate analysis, among patients of African origin, advanced fibrosis was independently associated with a significantly increased likelihood of bulevirtide use (OR = 5.1; 95% CI [2.16–12.01]; *p* < 0.0001). Conversely, no association between fibrosis stage and bulevirtide use was observed in patients born in other countries (OR = 0.86; 95% CI [0.36–2.16]; *p* = 0.7537). Neither sex nor age demonstrated a significant relationship with bulevirtide exposure (see Supplementary Materials Table S3).

### 3.10. Hepatitis Delta Treatments According to the Stage of Fibrosis

**Figure 11:**
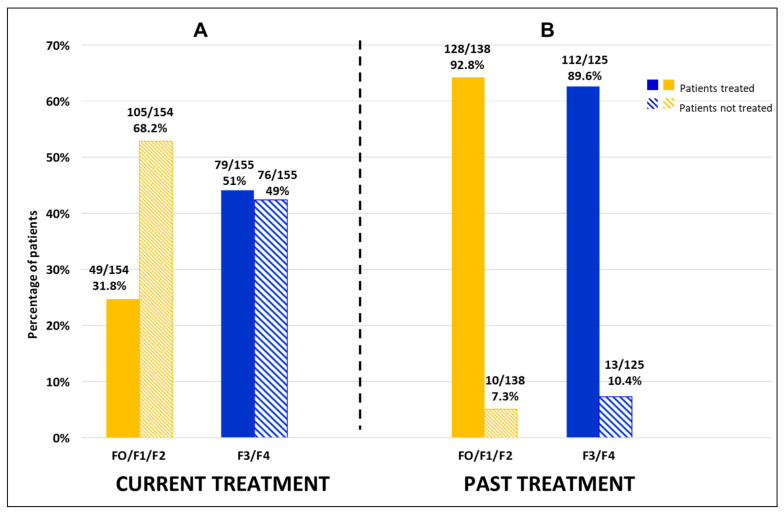
Distribution of current or past delta treatments according to the presence or absence of advanced fibrosis at the time of the questionnaire. F0/F1/F2 (N = 199); F3/F4 (N = 179). (**A**) current treatments. (**B**) past treatments.

Excepting patients with no treatment available, 31.8% (49/154) of patients without advanced fibrosis were on HDV and/or HBV treatment at the time of the survey (79.6% of them were on BLV and 10.2% were on PEG-IFN + BLV), and 92.8% (128/138) had previously received HDV and/or HBV treatment (64.1% of them PEG-IFN alone and 20.3% PEG-IFN + BLV). Meanwhile, 51% of the patients with advanced fibrosis (79/155) were receiving treatment at the time of the survey (72.2% of them were on BLV and 24.1% were on PEG-IFN + BLV), and 90% (112/125) had previously received therapy (50% of them had received PEG-IFN alone, 25.9% BLV + INF and 8% BLV alone) (Figure 11A,B).

In total, 222 patients received BLV, of whom 79 had no advanced fibrosis and 111 had advanced fibrosis; fibrosis data were unavailable for 32 patients.

## 4. Discussion

In this real-world study, 547 patients from mainland France who had at least one positive HDV RNA test in 2019 were contacted between 22 January 2024 and 22 July 2025, representing 14% of the 3800 estimated RNA-positive patients in metropolitan France [5]. We found demographic characteristics comparable to those reported in the French NRC involving patients managed in specialized reference centers [6]: young age and birth in endemic countries, including African and Eastern European countries and Mongolia. In France, most migrants have access to dedicated reception centers where systematic screening for viral hepatitis and HIV is performed using rapid diagnostic tests or venipuncture. Although they lack residence permits, they are granted State Medical Aid (AME) through social services, ensuring healthcare access. Hepatitis delta testing is routinely conducted upon the detection of HBsAg positivity; however, ongoing staff education is required to enhance screening awareness. An analogous strategy is recommended for people who inject drugs in addiction treatment centers, accounting for their greater sociobehavioral instability.

The estimated sexual risk, which was 5% for men who have sex with men in the NRC study, was not reported during the interviews, likely due to the difficulty of addressing this topic in a single phone call. The second route of transmission was drug use. The prevalence of HIV coinfection was comparable to that observed in the NRC study, whereas HCV coinfection was much lower. Explanations for this include patients’ possible unawareness of an HCV coinfection, with a spontaneous cure or a previous successful HCV treatment.

Patients identified based on HDV RNA test prescription were, unsurprisingly, mainly managed by a hepato-gastroenterology or infectious disease specialist, with at least biannual monitoring for 70% of them. In the first part of the Delta Describe study [11] focusing on SNDS data (2016–2022 period), we showed that these specialists, who are particularly involved in the management of delta hepatitis, were the main prescribers of HDV RNA tests [18].

The year of diagnosis was collected for 88.1% of patients. Patients also demonstrated a strong understanding of the severity of their condition and were able to articulate their complications (Table 2). The fibrosis score, which was almost uniformly assessed using FibroScan^®^, was accurately reported by 69.1% of the patients. Unsurprisingly, nearly half of the patients had advanced fibrosis and/or cirrhosis, and these results are consistent with the 45.2% figure reported in the French ANRS CO22 HEPATHER cohort [20]. The prevalence of advanced fibrosis was lower among patients of African ancestry, regardless of age, as in the CNR study [6].

In our study, half of the patients reported having previously undergone a liver biopsy. The accuracy of FibroScan^®^ has been validated in hepatitis delta by two recent publications, showing that advanced fibrosis can be ruled out in more than 90% of cases [18]. Moreover, the Baveno VII criteria have also been validated for the assessment of clinically significant portal hypertension in this population [21]. In the future, routine FibroScan^®^ implementation should facilitate patient management.

Of all the patients surveyed (excluding patients with only liver transplantation), 14.3% reported severe complications, principally portal hypertension and HCC events. Globally, the results seem better than in the French Deltavir cohort, which reported 48.8% of patients having cirrhosis, 24.2% having had one or more episodes of decompensation and 9.2% having HCC [6], at the end of a median 3.0-year [0.8–7.2] follow-up. Notably, this was before the use of BLV became widespread. However, the complication rate remains higher than in hepatitis B: in a case–control study of HBV-HDV patients compared to HBV patients in the French ANRS CO22 HEPATHER cohort, the incidence rates of decompensated cirrhosis, HCC, and transplantation were 10, 5, and 10 times higher in hepatitis delta than in hepatitis B, respectively [20].

Among patients undergoing treatment for HDV at the time of the survey, 84.8% were receiving NUCs, demonstrating satisfactory adherence to French and international guidelines [9,10]. Nevertheless, only two-thirds of patients with a known HBV viral load had controlled HBV DNA (below the detection threshold), which could be due to either insufficient adherence to nucleos(t)ide analogs, resistance to entecavir (for example, in patients previously treated with lamivudine), or the high persistence of cccDNA in the majority of patients [22].

In the population undergoing HBV and/or HDV treatment, overall, 40.6% (222/547) of patients had received or were receiving BLV with or without PEG-IFN, with their combination being recommended for compensated disease by the HAS. This opportunity was made possible thanks to the drug being granted marketing authorization in France and its reimbursement. However, BLV treatment was proposed more often to patients at advanced fibrosis stages (stages F3 and F4), particularly to those of African origin. Finally, 2.8% of the patients treated were included in a clinical therapeutic trial or benefited from compassionate use programs. Enhancing access to treatment, whatever the fibrosis score, should allow for a drastic reduction in long-term complications [8]. In a recent European retrospective multicenter real-world study, it was demonstrated that at week 96 of BLV treatment, the cumulative risks of de novo HCC and cirrhosis decompensation were only 3.0% (95% CI 2–6%) and 2.8% (95% CI 1–5%), respectively [15].

Finally, among patients who had at least one positive HDV RNA test in 2019, 47.1% had a negative HDV RNA test at the time of the interview, and 40.6% had received or were receiving BLV. In this vulnerable and unstable population, the relatively high percentage of HDV RNA-negative cases suggests a high adherence and long-term persistence rate to bulevirtide, as described in the Barodelta study [23].

### 4.1. Limitations of the Study

Only patients from metropolitan France were included in this study. The profile of patients from overseas territories is highly likely to be different from our cohort. Moreover, 30% of the HDV RNA-positive population could not be interviewed because of a loss of contact during follow-up or their objection to being included in the study. As the data were obtained directly from the patients, they may be biased by subjectivity, although many of them could be verified through interviews with physicians. Fibrosis assessment using Fibroscan^®^ was not systematically obtained in the year of the hepatitis delta diagnosis.

A total of 10% of F0/F2 patients declared liver-related complications. The first hypothesis is that some fibrosis scores (F0/F1/F2) may have been underestimated because they were measured during antiviral treatment. The second hypothesis, as recently suggested, is that HDV also partially increases the risk of hepatic events, independently of underlying fibrosis. This reinforces the importance of anti-HDV treatments in limiting fibrosis, its complications, and the overall incidence of hepatic events [20].

Moreover, the total prevalence of liver complications may have been underestimated because transplanted patients were not always able to report the complications that led to transplantation.

Finally, data on the timing of delta treatment were not always available, and reasons for stopping bulevirtide treatment could not be identified.

### 4.2. Perspectives

Strengthening HDV screening among at-risk populations—particularly for migrants and people who inject drugs, with limited healthcare access—requires innovative outreach strategies. Organized HBV, HCV, and HIV screening programs, such as the previously reported “Scanvir” initiative using dedicated multidisciplinary sessions, provide a supportive framework [24]. Blot-based assays for anti-HDV antibodies and HDV RNA are under development. In parallel, an ongoing study is assessing the feasibility and cost-effectiveness of reflex HDV testing by clinical laboratories for newly identified HBsAg-positive cases.

## 5. Conclusions

This real-world study, conducted in metropolitan France, suggests that patients with hepatitis delta should have access to specialist care and effective treatments such as bulevirtide. This can impact the prognosis of the disease, regardless of the degree of fibrosis. The data provided by patients, compared with those provided by the attending specialist, when possible, demonstrate that patients understand their pathology and related risks well. This study also shows that, since 2019, the systematic prescription of nucleoside analogs has mostly been carried out in accordance with international recommendations.

## Figures and Tables

**Table 1:** Data collected.

Place of birth Potential route of contamination Date of their delta infection diagnosis HCV and/or HIV coinfection General practitioner or specialist primarily responsible for the patient’s care Level of fibrosis Method of fibrosis assessment: Fibroscan^®^, liver biopsy, biological non-invasive tests Complications of hepatitis delta HDV RNA and HBV DNA status at the time of the questionnaire HBV treatment history HDV treatment history

**Table 2:** Liver complications (decompensated cirrhosis and HCC) reported by patients.

Complications Reported by 67 Patients	N = 73
Ascites	N = 11
Hepatorenal syndrome	N = 1
Esophageal varices	N = 20
Gastrointestinal bleeding	N = 3
Portal hypertension	N = 10
Hyperbilirubinemia	N = 5
Cirrhosis decompensation	N = 3
Fulminant hepatitis	N = 1
HCC	N = 19

## Data Availability

The data on which this article is based cannot be made public due to the confidentiality of the individuals who participated in the study, even though all data have been anonymized.

## Supplementary Materials

The following supporting information can be downloaded at: https://www.mdpi.com/article/10.3390/v18040424/s1, Table S1: Relationship between level of liver fibrosis and clinical and demographic variables; Table S2: Relationship between patients’ complications and demographic and clinical variable; Table S3: Relationship between past or current use of bulevirtide and clinical and demographic variables.

## Abbreviations

Ab	Antibody
AG	Antigen
CNIL	National Commission on Informatics and Liberty
DNA	Deoxyribonucleic Acid
eCRF	Electronic Case Report Form
BLV	Bulévirtide
HAS	French National Authority for Health
HBV	Hepatitis B virus
HCC	Hepatocellular carcinoma
HDV	Hepatitis delta virus
HCV	Hepatitis C virus
HIV	Human immunodeficiency virus
IQR	Interquartile Range
NUC	Nucleoside analogues treatments
NRC	National Reference Center
PCR	Polymerase chain reaction
PEG-IFN	Pegylated interferon
RNA	Ribonucleic acid
SNDS	French National Health Data System
WHO	World Health Organization
