# Supplementary material for: Delta Describe, the French Collaborative Project: The Profile and Management of Hepatitis Delta Patients in Metropolitan France
Source: Viruses. 2026 Mar 31;18(4):424. doi: 10.3390/v18040424 (PMC13120407; doi:10.3390/v18040424)
Supplement: Supplementary file 1 [file viruses-18-00424-s001.zip › viruses-4186834-supplementary.pdf]

| *Level of liver fibrosis* | F0/F1/F2 (n = 199) n (%) | F3/F4 (n = 179) n (%) | Univariate model | | | Multivariate model | | |
|---|---|---|---|---|---|---|---|---|
| | | | OR brut [IC95%] | p-value | p-value globale | OR brut [IC95%] | p-value | p-value globale |
| **Sex** | | | | | 0. 1201 | | | |
| - *Female* | 81 (40.7%) | 59 (33%) | - | - | | | | |
| - *Male* | 118 (59.3%) | 120 (67%) | 1.40 [0.92 ; 2.13] | 0.1201 | | | | |
| **Age** | | | | | 0.0071 | | | **See Interaction** |
| - *< 40 years* | 82 (41.2%) | 50 (27.9%) | - | - | | - | - | |
| - *≥ 40 years* | 117 (58.8%) | 129 (72.1%) | 1.81 [1.17 ; 2.78] | 0.0071 | | | | |
| **Migrants' country of birth** | | | | | **<0.0001** | | | **See Interaction** |
| - *Not native of Africa (Eurasia or Guyana origin)* | 66 (35.3%) | 93 (60%) | - | - | | | | |
| - *Africa origin* | 121 (64.7%) | 62 (40%) | 0.36 [0.23 ; 0.56] | **<0.0001** | | | | |
| - *Missing data* | 12 (.%) | 24 (.%) | | | | | | |
| **INTERACTION :** | | | | | | | | |
| **Between age and migration:** | | | | | | | | **0.0214** |
| - *Age < 40 years and African origin (vs - Not native of Africa) impact level of fibrosis* | | | | | | 0.18 [0.08 ; 0.39] | **<0.0001** | |
| - *Age ≥ 40 years and African origin (vs Not native of Africa) impact level of fibrosis* | | | | | | 0.54 [0.32 ; 0.93] | **0.0264** | |

**Table S1: Relationship between level of liver fibrosis and clinical and demographic variables**

| *Complications* | Yes (n = 102) n (%) | No (n = 366) n (%) | **Univariate model** | | | **Multivariate model** | |
|---|---|---|---|---|---|---|---|
| | | | **OR [IC95%]** | **p-value** | **Global p-value** | **OR [IC95%]** | **p-value** |
| **Sex** | | | | | 0.6671 | | |
| - *Female* | 40 (39.2%) | 135 (36.9%) | - | - | | | |
| - *Male* | 62 (60.8%) | 231 (63.1%) | 0.91 [0.58 ; 1.4] | 0.6671 | | | |
| **Age** | | | | | **<0.0001** | | |
| - *< 40 ans* | 20 (19.6%) | 133 (36.3%) | - | - | | | |
| - *≥ 40 ans* | 82 (80.4%) | 233 (63.7%) | 2.3 [1.37; 3.99] | **0.0018** | | | |
| **Migrants' country of birth** | | | | | **0.0002** | | |
| - *Africa* | 34 (39.5%) | 211 (62.1%) | - | - | | | |
| - *Not native of Africa* | 52 (60.5%) | 129 (37.9%) | 0.4 [0.25 ; 0.65] | **0.0002** | | | |
| - *Missing data* | 16 (.%) | 26 (.%) | | | | | |
| **Level of liver fibrosis** | | | | | **< 0.0001** | | |
| - *F0/F1/F2* | 19 (28.8%) | 171 (60.2%) | - | - | | | |
| - *F3/F4* | 47 (71.2%) | 113 (39.8%) | 3.74 [2.09 ; 6.71] | **< 0.0001** | | 3.74 [2.09 ; 6.71] | **< 0.0001** |
| - *Missing data* | 36 (.%) | 82 (.%) | | | | | |

**Table S2: Relationship between patients' complications and demographic and clinical variables**

| *Current or past bulevirtide use* | Yes (n = 222) n (%) | No (n = 101) n (%) | Univariate model | | | Multivariate model | | |
|---|---|---|---|---|---|---|---|---|
| | | | OR [IC95%] | p-value | Global p-value | OR brut [IC95%] | p-value | Global p-value |
| **Sex** | | | | | 0.6847 | | | |
| - *Female* | 74 (33.3%) | 36 (35.6%) | - | - | | | | |
| - *Male* | 148 (66.7%) | 65 (64.4%) | 1.11 [0.68 ; 1.82] | 0.6847 | | | | |
| **Age** | | | | | 0.7039 | | | |
| - *< 40 years* | 70 (31.5%) | 34 (33.7%) | - | - | | - | - | |
| - *≥ 40 years* | 152 (68.5%) | 67 (66.3%) | 1.1 [0.67 ; 1.82] | 0.7039 | | | | |
| **Migrants' country of birth** | | | | | **0.0014** | | | **See interaction** |
| - *Not native of Africa* | 113 (55.7%) | 30 (34.9%) | - | - | | | | |
| - *Africa* | 90 (44.3%) | 56 (65.1%) | 0.43 [0.25 ; 0.72] | **0.0014** | | | | |
| - *Missing data* | 19 (.%) | 15 (.%) | | | | | | |
| **Level of liver fibrosis** | | | | | **0.0009** | | | **See interaction** |
| - *F0/F1/F2* | 79 (41.6%) | 55 (63.2%) | - | - | | | | |
| - *F3/F4* | 111 (58.4%) | 32 (36.8%) | 2.42 [1.43 ; 4.07] | **0.0009** | | | | |
| - *Missing data* | 32 (.%) | 14 (.%) | | | | | | |
| **INTERACTION :** | | | | | | | | |
| **Between migrants' origin and liver fibrosis level** | | | | | | | | **0.0022** |
| - *African origin and fibrosis (F3/F4 vs F0/F1/F2)* | | | | | | 5.1 [2.16 ; 12.01] | **<0.0001** | |
| - *Not native of Africa and fibrosis (F3/F4 vs F0/F1/F2)* | | | | | | 0.86 [0.36 ; 2.16] | 0.7537 | |

**Table S3: Relationship between past or current use of bulevirtide and clinical and demographic variables**
